# Factors influencing health care use by health insurance subscribers and medical aid beneficiaries: a study based on data from the Korea welfare panel study database

**DOI:** 10.1186/s12889-020-09073-x

**Published:** 2020-07-20

**Authors:** Na Young Kong, Dong Hee Kim

**Affiliations:** 1 Health Insurance Review & Assessment Service, Wonju, Republic of Korea; 2 College of Nursing, Pusan National University, Beomeo-ri, Mulgeum-eup, Yangsan-si, Gyeongsangnam-do 626–870 Republic of Korea

**Keywords:** Health care, Health care use, Health care services, Health insurance, Medical aid

## Abstract

**Background:**

The use of health care services is influenced by various factors, including demographic, social, economic, and health status factors. This study aimed to identify the factors that influence health care use in health insurance subscribers and medical aid beneficiaries in Korea.

**Methods:**

A total of 11,793 subjects were identified, including 10,838 health insurance subscribers and 955 medical aid beneficiaries, using the Korea Welfare Panel Study database. The data were analysed by percentage, t-test, and multiple regression using SPSS 20.0.

**Results:**

Medical aid beneficiaries had 13.51 more days of outpatient visits and 8.38 more days of hospitalization compared with health insurance subscribers. Factors affecting the frequency of outpatient visits for health insurance subscribers were gender, age, household type, education level, income level, administrative district, perceived health status, chronic disease, and disability. These factors accounted for 19.8% of explanation (*p* < .001). Whereas, gender, household type, administrative district, perceived health status, and chronic disease were identified as factors influencing outpatient frequency for medical aid beneficiaries. These factors accounted for 11.2% of explanation (*p* < .001). For health insurance subscribers, factors affecting the length of hospitalization were gender, public pension status, place of residence, administrative district, economic activity, income level, perceived health status, and disability status. These factors accounted for 7.2% of explanation (*p* < .001). While, factors affecting the length of hospitalization for medical aid beneficiaries were accounted for by 3.4% (*p* < .001). Gender and perceived health status were identified as factors influencing the length of hospitalization of medical aid beneficiaries.

**Conclusions:**

There were differences between medical aid beneficiaries and health insurance subscribers in health care use and influencing factors. Future management programs should take into consideration the specific factors that influence the use of health care services in health insurance subscribers and medical aid beneficiaries.

## Background

South Korea has been ranked as first among the Organisation for Economic Co-operation and Development (OECD) countries in health care access. The country’s colorectal cancer survival rate was at 72.8%, which is the OECD’s highest rate, ahead of Denmark’s 55.5% and the United Kingdom’s 54.5%. Korea had the second highest cervical cancer survival rate at 76.8%, ahead of Germany’s 64.5% and the United States’ 62.2% [1]. The country’s medical insurance system is divided into the National Health Insurance system and the Medical Aid system. The National Health Insurance system plays an important role in improving national health by providing insurance benefits for medical expenses and health promotion. The Medical Aid system provides medical care services such as examination, and treatment for medical problems, such as illness, injury, and childbirth to people with low-income [2]. Among the total Korean population in 2015, the number of people covered by health insurance was 50.49 million, accounting for 97% of the total population, and the number of beneficiaries was 1.55 million, accounting for 3% of the total population. Health insurance subscribers pay 20% (50% of total bills) for hospitalization and 30 ~ 60% for outpatient visiting based on a standard fee system when using medical services. While medical aid beneficiaries pay 10% or free for hospitalization and a fixed amount (1000 to 2000 won) for outpatient visiting when using medical services [3].

Since the initiation of the medical insurance system, the total cost of health insurance and the total cost of medical benefits have been increasing. Total health insurance expenses increased 1.4 times in 2015 (from about 32 trillion won in 2007 to about 47 trillion won). Total medical expenses also increased, with growth rates similar to that of health insurance expenses, from 4.2 trillion won to 6 trillion won during the same period [3]. However, health care service use and number of hospitalization days in medical aid beneficiaries were about four times higher than that in health insurance subscribers, and the medical expenses per person in medical aid beneficiaries were about 3.6 times higher than that in health insurance subscribers [4]. It has been argued that medical aid beneficiaries’ overuse of free medical services has been deemed one of the main causes for the rise in medical costs. We need to identify the factors influencing health care use by health insurance subscribers and compare the factors to those medical aid beneficiaries.

The use of health care services is influenced by various factors. It is influenced not only by disease, but also by demographic, social, economic, and health status factors and these factors differ between medical aid beneficiaries and health insurance subscribers [5]. However, previous studies have focused on individual factors, such as socio-demographic characteristics [6], socio-economic characteristics [7], or specific age groups or people with specific diseases [8], and have used a cross-sectional design [7, 8]. Medical aid beneficiaries are older, experience more economic difficulty, and are more likely to be unemployed, compared with health insurance subscribers [9]. Members of this group have high levels of medical need because incidences of complex and chronic diseases are higher in this group than in health insurance subscribers. Medical aid beneficiaries have more difficulty utilizing effective and efficient medical services due to less ability to care for themselves compared with health insurance subscribers [10]. Differences in health care use between health insurance subscribers and medical aid beneficiaries appear to be closely related to socio-demographic factors, economic factors, and health status.

Previous studies simply compared the health care use of health insurance subscribers and medical aid beneficiaries, focusing on the expansion of coverage and the introduction of co-payments. These studies compared the total health care costs, inpatient care costs, outpatient care costs, medication days, and outpatient care costs between health insurance subscribers and medical aid beneficiaries without considering the general characteristics and health conditions of those who were insured and those receiving benefits. In addition, one limitation of that study was that the possible factors of health care use were analysed with a convenience sample of subjects.

It is necessary to examine health care use differences between different medical insurance types and the influencing factors in order to develop effective medical management policies. The purpose of this study was to analyse health care use behaviours and influencing factors in health insurance subscribers and medical aid beneficiaries. More specifically, this study shows the differences the number of outpatient visits and the length of hospitalization according to socio-demographic, socio-economic, and health status of subjects. In addition, we examine health care use status and the factors affecting health care use in health insurance subscribers and medical aid beneficiaries.

The 10th Korea Welfare Panel Study (2015) allocated about 50% of the total sample to low-income people who were less than 60% of median income. It added a questionnaire on economic activity participation status. In addition, because it includes the demographic, social, economic, and health status of the subjects, the characteristics of the subjects can be examined as well as the difference in the number of days of medical treatment and the difference in medical expenses.

## Methods

### Design

This study used a descriptive research design to examine health care use status and the factors affecting health care use in health insurance subscribers and medical aid beneficiaries by analysing secondary data using the 10th Korea Welfare Panel Study (2015) annually integrated data.

### Subjects

This study analysed the annual integrated data of the 10th Korea Welfare Panel Study (2015) jointly conducted by the Korea Institute for Health and Social Affairs and the Seoul National University Institute for Social Welfare. The data used in this study were obtained through the Korea Welfare Panel homepage (https://www.koweps.re.kr).

The sampling method of the 10th Korea Welfare Panel Study (2015) consisted of two stages. In the first stage, 517 survey areas were selected from the population census data to investigate household income and economic activity status of household members. In the second stage, the stratified double sampling method was used. The population was 7188 households among 242 municipalities in 16 cities, of which 6914 households participated in the survey (96.19% participation rate). With the distribution of samples, 3500 households with low-income households with a median income of less than 60% and 3500 households with a median income of 60% or more were extracted. The survey period was from March 2, 2015, to June 12, 2015.

### Measurements

Measurements included health care service use, socio-demographic factors, socio-economic factors and health status (supplementary 1). Factors reported in previous studies as variables affecting the number of outpatient clinic visits and the number of hospitalization days were included.

1. **Health care service use.**

Health care service use was assessed with the number of outpatient clinic visits and number of hospitalization days in 2014.

2) **Socio-demographic factors.**

Socio-demographic characteristics included gender, age, spouse, household type, education, private health insurance, public pension, residential area, and administrative district.

3) **Socio-economic factors.**

The socio-economic factors examined in this study included financial activity participation and low-income household status.

4) **Health status.**

Health status characteristics examined in the study included perceived health status, chronic disease, and disability. Chronic diseases were categorized by administration of medication or medical treatment. Absence of autism disorders, epilepsy disorders, mental disorders, kidney disorders, heart disorders, respiratory disorders, liver disorders, facial disorders, and ostomy disorders was classified as non-disabled.

### Data collection

The study protocol was approved by the Institutional Review Board. Before collecting the data, information and guideline sheets were distributed by mail twice, and then investigators visited the households and surveyed the subjects. The investigators used a chronological interview method in which the subjects responded using the computer-assisted personal interview program. The 10th Korea Welfare Panel Study (2015) was conducted for 103 days (from March 2, 2015, to June 12, 2015).

### Data analysis

Data were analysed using the SPSS/WIN 20.0 program (IBM, Armonk, NY, USA). Socio-demographic characteristics, socio-economic characteristics, health status, and health care use were analysed by frequency, percentage, and mean and standard deviation. The differences in health care use based on socio-demographic characteristics, socio-economic characteristics, and health status were analysed by means, standard deviation, and t-test or ANOVA. Stepwise multiple regression analysis was performed by inputting significant variables based on the t-test or ANOVA as independent variables and the number of outpatient visits or the length of hospitalization as the dependent variables. The significance level used for data analysis was *p* ≤ 0.05.

## Results

### Socio-demographic, socio-economic, and health status characteristics of subjects

The socio-demographic, socio-economic, and health status characteristics of the subjects are shown in Table 1. The total number of subjects was 11,793, with 10,838 health insurance subscribers (91.9%) and 995 (8.1%) medical aid beneficiaries.

**Table 1:** Socio-demographic, socio-economic, and health status characteristics of subjects (*N* = 11,793)

Characteristics		Total n (%)	Health insurance subscribers (*n* = 10,838) n (%)	Medical aid beneficiaries (*n* = 955) n (%)
Gender	Men	6650 (44.5)	5955 (54.9)	360 (37.7)
	Women	5243 (55.5)	4883 (45.1)	595 (62.3)
Age	≤19	181 (1.5)	119 (1.1)	52 (6.5)
	20–29	393 (3.3)	343 (3.2)	50 (5.2)
	30–39	1336 (11.3)	1314 (12.1)	22 (2.3)
	40–49	2087 (17.7)	1990 (18.4)	97 (10.2)
	50–59	2023 (17.3)	1856 (17.1)	167 (17.5)
	60–69	1995 (16.9)	1829 (16.8)	166 (17.4)
	≥70	3778 (32.0)	3387 (31.3)	391 (40.9)
Having a spouse	Yes	8107 (68.7)	7844 (72.4)	263 (27.5)
	No	3686 (31.3)	2994 (27.6)	692 (72.5)
Household type	Single	2010 (17.0)	1642 (15.2)	368 (38.5)
	Non-single	9783 (83.0)	9196 (84.8)	587 (61.5)
Education	None	1151 (9.7)	968 (8.9)	183 (19.2)
(Graduation)	Primary school	2707 (23.0)	2409 (22.2)	298 (31.2)
	Secondary school	4969 (42.1)	4583 (42.3)	386 (40.4)
	College	2966 (25.2)	2878 (26.6)	88 (9.2)
Private health	Join	6435 (54.6)	6214 (57.3)	221 (23.1)
Insurance	Do not join	5358 (45.4)	4624 (42.7)	734 (76.9)
Public pension	Join	6170 (52.3)	6077 (56.1)	93 (9.7)
	Do not join	5623 (47.7)	4761 (43.9)	862 (90.3)
Residential area	City	9395 (79.7)	8595 (79.3)	800 (83.8)
	Rural	2398 (20.3)	2243 (20.7)	155 (16.2)
Administrative	Capital	4184 (35.5)	3887 (35.9)	297 (31.1)
Area	Non-capital	7609 (64.5)	6951 (64.1)	658 (68.9)
Financial activity	Yes	10,344 (87.7)	9726 (89.7)	618 (64.7)
Participation	No	1449 (12.3)	1112 (10.3)	337 (35.3)
Low-income	Yes	7498 (63.6)	3411 (31.5)	884 (92.6)
household	No	4295 (36.4)	7427 (68.5)	71 (7.4)
Perceived health	Very good	1060 (9.0)	1035 (9.5)	25 (2.6)
Status	Good	5246 (44.6)	5047 (46.6)	199 (20.8)
	Neutral	2789 (23.6)	2529 (23.4)	260 (27.2)
	Poor	2480 (21.0)	2050 (18.9)	430 (45.0)
	Very poor	218 (1.8)	177 (1.6)	41 (4.4)
Presence of	Yes	6853 (58.1)	6089 (56.2)	764 (80.0)
Chronic illness	No	4940 (41.9)	4749 (43.8)	191 (20.0)
Disability	Yes	1331 (11.4)	1031 (9.6)	300 (31.7)
	No	10,390 (88.6)	9745 (90.4)	645 (68.3)

### Average number of outpatient visits and the length of hospitalization

Of the total 11,793, the number of people who visited outpatient and admitted over the past year was 10,219 and 1608 respectively. Of the total 10,838 health insurance subscribers, 9329 (86.1%) used outpatient visits and 1435 (13.2%) used hospitalization. And 890 (93.2%) out of 955 medical aid beneficiaries visited outpatient visits and 173(18.1%) used hospitalization. The average number of outpatient visits over the past year was 20.64 days for health insurance subscribers and 34.15 days for medical aid beneficiaries. The average length of hospitalization in the past year was 21.58 days for health insurance subscribers and 29.97 days for medical aid beneficiaries (Table 2).

**Table 2:** Average number of outpatient visits and the length of hospitalization (*N* = 11,793)

		Health insurance subscribers (*n* = 10,838)	Medical aid beneficiaries (*n* = 955)	Total
Outpatient visits	People n (%)	9329 (86.1)	890 (93.2)	10,219 (86.7)
	Days	20.64	34.15	
Hospitalization	People n (%)	1435 (13.2)	173 (18.1)	1608 (13.6)
	Days	21.58	29.97	

### The number of outpatient visits according to socio-demographic, socio-economic, and health status characteristics

For health insurance subscribers, there were differences in the number of outpatient visits according to gender (*p* < .001), age (*p* < .001), having a spouse (*p* < .001), household type (*p* < .001), education level (*p* < .001), private health insurance (*p* < .001), public pension status (*p* < .001), residence (*p* < .001), administrative district (*p* < .001), financial activity participation (*p* < .001), income level (*p* < .001), perceived health status (*p* < .001), chronic disease (*p* < .001), and disability (*p* < .001).

For medical aid beneficiaries, there were differences in the number of outpatient visits according to gender (*p* < .001), household type (*p* < .001), education level (*p* = .015), administrative district (*p* = .001), financial activity participation (*p* = .010), perceived health status (*p* < .001), chronic disease (*p* < .001), and administrative area (*p* < .001) (Table 3).

**Table 3:** The number of outpatient visits according to socio-demographic, socio-economic, and health status of subjects (*N* = 11,793)

Characteristics		Health insurance subscribers (*n* = 10,838) M ± SD	t or F(p)	Medical aid beneficiaries (*n* = 955) M ± SD	t or F(p)
Gender	Men	15.61 ± 22.61	−15.13	27.25 ± 36.17	−4.01
	Women	24.38 ± 33.32	(<.001)	38.18 ± 43.83	(<.001)
Age	≤19	7.93 ± 8.20	7.03	10.15 ± 11.22	0.41
	20–29	7.81 ± 12.04	(<.001)	12.18 ± 21.83	(.814)
	30–39	7.81 ± 8.75		30.88 ± 38.59	
	40–49	8.77 ± 9.85		23.75 ± 29.25	
	50–59	14.58 ± 20.40		35.20 ± 50.24	
	60–69	22.14 ± 28.66		39.08 ± 43.13	
	≥70	33.08 ± 38.39		38.83 ± 40.87	
Having a spouse	Yes	17.81 ± 26.09	13.18	31.78 ± 37.90	1.12
	No	28.09 ± 36.14	(<.001)	35.08 ± 42.80	(.262)
Household	Single	34.30 ± 39.93	−15.37	41.30 ± 45.99	−4.12
Type	Non-single	17.96 ± 26.25	(<.001)	29.28 ± 37.38	(<.001)
Education	None	37.09 ± 40.58	4.98	40.61 ± 44.83	2.44
(Graduation)	Primary	31.03 ± 37.02	(<.001)	38.88 ± 43.62	(.015)
	Secondary	16.64 ± 23.84		30.26 ± 39.78	
	College	10.04 ± 14.38		17.41 ± 20.44	
Private health	Join	14.30 ± 21.12	22.40	29.94 ± 47.37	1.62
Insurance	Do not join	28.32 ± 35.84	(<.001)	35.35 ± 39.62	(.106)
Public pension	Join	16.36 ± 24.37	14.90	33.98 ± 50.12	0.04
	Do not join	25.62 ± 33.97	(<.001)	34.17 ± 40.51	(.967)
Residential	City	19.01 ± 27.65	8.91	33.42 ± 39.23	1.01
Area	Rural	26.48 ± 34.65	(<.001)	38.07 ± 51.88	(.313)
Administrative	Capital	17.10 ± 25.04	9.17	27.81 ± 32.18	3.46
Area	Non-capital	22.57 ± 31.06	(<.001)	36.99 ± 44.77	(.001)
Financial activity	Yes	18.84 ± 26.43	11.23	31.33 ± 39.86	2.58
Participation	No	34.72 ± 44.99	(<.001)	38.91 ± 43.76	(.010)
Low-income	Yes	31.26 ± 37.69	22.18	34.52 ± 41.60	0.92
Household	No	15.14 ± 22.42	(<.001)	29.69 ± 40.14	(.360)
Perceived health	Very good	7.05 ± 10.40	8.13	8.27 ± 10.25	6.18
Status	Good	11.37 ± 14.32	(<.001)	18.72 ± 25.68	(<.001)
	Neutral	26.15 ± 33.05		32.09 ± 38.88	
	Poor	36.40 ± 40.17		39.70 ± 44.14	
	Very poor	32.99 ± 41.71		58.79 ± 58.68	
Presence of	Yes	28.12 ± 33.81	−45.44	38.52 ± 43.24	−16.21
Chronic illness	No	6.94 ± 9.55	(<.001)	8.87 ± 10.75	(<.001)
Disability	Yes	31.52 ± 41.73	−8.87	34.05 ± 40.19	0.05
	No	19.36 ± 27.52	(<.001)	34.20 ± 42.42	(.959)

### Length of hospitalization according to socio-demographic, socio-economic, and health status characteristics

For health insurance subscribers, the length of hospitalization was statistically different according to gender (*p* < .001), age (*p* < .001), having a spouse (*p* < .001), household type (*p* < .001), education level (*p* < .001), economic activity participation (*p* < .001), income level (*p* < .001), private health insurance (*p* < .001), public pension status (*p* < .001), residence (*p* < .001), administrative district (*p* < .001), perceived health status (*p* < .001), chronic disease (*p* < .001), and disability (*p* < .001).

For medical aid beneficiaries, there were statistical differences in length of hospitalization according to gender (*p* = .011), economic activity participation (*p* = .024), private medical insurance (*p* = .029), perceived health status (*p* = .043), and chronic disease (*p* < .001) (Table 4).

**Table 4:** The length of hospitalization according to socio-demographic, socio-economic, and health status of subjects (*N* = 11,793)

Characteristics		Health insurance subscribers (*n* = 10,838) Days M ± SD	t or F(p)	Medical aid beneficiaries (*n* = 955) Days M ± SD	t or F(p)
Gender	Men	26.55 ± 46.57	3.69	45.51 ± 70.20	2.60
	Women	18.47 ± 27.68	(<.001)	21.06 ± 34.05	(.011)
Age	≤19	0.57 ± 3.98	3.01	5.21 ± 38.09	1.59
	20–29	1.11 ± 6.09	(<.001)	1.86 ± 5.80	(.205)
	30–39	0.81 ± 3.44		6.91 ± 26.05	−0.35
	40–49	1.28 ± 10.74		5.49 ± 31.50	(.725)
	50–59	1.34 ± 7.31		5.71 ± 26.42	
	60–69	2.94 ± 13.86		8.57 ± 33.62	
	≥70	5.62 ± 22.55		4.37 ± 14.16	
Having a spouse	Yes	2.54 ± 14.75	3.37	5.89 ± 28.44	−0.35
	No	3.68 ± 15.98	(.001)	5.25 ± 23.17	(.725)
Household	Single	3.85 ± 14.44	−2.99	6.56 ± 25.87	−1.12
Type	Non-single	2.68 ± 15.22	(.003)	4.72 ± 23.97	(.263)
Education	None	5.92 ± 22.78	1.32	6.05 ± 26.72	1.62
(Graduation)	Primary	5.12 ± 21.49	(<.001)	5.05 ± 19.57	(.183)
	Secondary	2.10 ± 12.13		5.44 ± 28.72	
	College	1.14 ± 7.30		5.38 ± 15.83	
Private health	Join	1.57 ± 7.81	9.25	3.29 ± 11.47	2.18
Insurance	Do not join	4.59 ± 21.16	(<.001)	6.07 ± 27.46	(.029)
Public pension	Join	1.73 ± 9.00	8.13	5.23 ± 30.13	0.08
	Do not join	4.30 ± 20.31	(<.001)	5.45 ± 24.09	(.934)
Residential	City	2.25 ± 12.14	5.78	5.06 ± 24.61	1.06
Area	Rural	5.17 ± 23.07	(<.001)	7.35 ± 25.26	(.289)
Administrative	Capital	1.98 ± 10.53	5.16	3.87 ± 19.71	1.47
Area	Non-capital	3.35 ± 17.13	(<.001)	6.13 ± 26.66	(.143)
Financial activity	Yes	1.84 ± 10.06	9.33	3.96 ± 21.42	2.27
Participation	No	11.78 ± 35.39	(<.001)	8.12 ± 29.69	(.024)
Low-income	Yes	4.16 ± 17.99	5.50	5.60 ± 25.44	0.74
Household	No	2.26 ± 13.54	(<.001)	3.34 ± 12.73	(.460)
Perceived health	Very good	0.51 ± 4.03	20.36	0.28 ± 1.40	2.48
Status	Good	0.86 ± 4.82	(<.001)	1.14 ± 7.10	(.043)
	Neutral	2.07 ± 8.72		2.19 ± 10.76	
	Poor	7.49 ± 25.51		8.90 ± 34.02	
	Very poor	31.30 ± 58.37		13.54 ± 28.08	
Presence of	Yes	4.36 ± 18.82	−12.85	6.60 ± 2746	−5.68
Chronic illness	No	0.94 ± 7.77	(<.001)	0.76 ± 3.61	(<.001)
Disability	Yes	6.87 ± 29.50	−4.87	8.21 ± 36.90	−1.83
	No	2.36 ± 12.01	(<.001)	4.14 ± 16.32	(.068)

### Influencing factors of the number of outpatient visits and the length of hospitalization

Factors affecting the average number of outpatient visits and the length of hospitalization are shown in Table 5. For health insurance subscribers, Durbin-Watson’s value was 1.82, the tolerance limit was between 0.40 and 0.92, and the variation inflation factor (VIF) was 1.09 to 2.56. Factors affecting the frequency of outpatient visits were gender (*p* < .001), age (*p* < .001), household type (*p* < .001), education level (*p* < .001), administrative district (*p* < .001), income level (*p* = .001), perceived health status (*p* < .001), chronic disease (*p* < .001), and disability(*p* < .001). These factors accounted for 19.8% of explanation (*p* < .001).

**Table 5:** Influencing factors of the number of outpatient visits and the length of hospitalization (*N* = 11,793)

	Number of outpatient visits				Length of hospitalization		
Health insurance subscribers							
(*n* = 10,838)		B	β	p	B	β	p
Gender	Women	6.02	0.10	<.001	−1.19	−0.04	<.001
Age		1.27	0.07	<.001	−0.09	−0.01	.516
Having a spouse	Yes	−0.38	−0.01	.669	−0.57	−0.02	.184
Household type	Single	5.35	0.07	<.001	−0.78	−0.02	.143
Education		−1.73	−0.05	<.001	−0.01	0.00	.954
Private health insurance	Join	−0.17	0.00	.812	0.45	0.02	.199
Public pension	Join	0.85	0.01	.192	−1.27	−0.04	<.001
Residential area	City	−0.02	0.00	.973	−1.88	−0.05	<.001
Administrative area	Capital	−2.64	−0.04	<.001	− 0.78	−0.03	<.001
Economic activity	Yes	−0.94	−0.01	.332	−6.48	−0.13	<.001
Low-income household	No	−2.41	−0.04	.001	2.07	0.07	<.001
Perceived health status		4.80	0.16	<.001	2.98	0.19	<.001
Chronic disease	Yes	10.19	0.16	<.001	−0.33	−0.01	.368
Disability	Yes	3.74	0.04	<.001	1.41	0.03	.004
R		.446				.269	
Adjusted R-square		.197				.072	
Durbin-Watson		1.822				1.944	
P		<.001				<.001	
Medical aid beneficiaries							
(*n* = 955)		B	β	p	B	β	p
Gender	Women	8.87	0.10	.002	−4.42	−0.09	.007
Household type	Single	6.40	0.08	.024			
Education		−1.03	−0.02	.534			
Private health insurance	Yes	–	–	–	0.05	−0.00	.981
Administrative area	Capital	−9.57	−0.11	.001	–	–	–
Economic activity	Yes	−2.27	−0.03	.427	−1.89	−0.04	.282
Perceived health status		5.84	0.12	.001	3.55	0.13	.001
Chronic disease	Yes	19.19	0.16	<.001	1.07	0.02	.660
R		.333				.177	
Adjusted R-square		.111				.031	
Durbin-Watson		2.000				2.009	
P		<.001				<.001	

^a^ Dummy coded:1 = Women, having a spouse, single household, no low-income household, join private health insurance, join public health pension, city residential area, capital administrative area, have chronic disease and have disability

Whereas, Durbin-Watson’s statistic was 2.00, the tolerance limit was between 0.70 and 0.99, and the VIF was from 1.07 to 1.41 for medical aid beneficiaries. The influencing factors for the number of outpatient visits were accounted for by 11.1% (*p* < .001). In detail, factors such as gender (*p* = .002), household type (*p* = .024), administrative district (*p* = .001), perceived health status (*p* = .001), and chronic disease (*p* < .001) were identified as factors influencing outpatient frequency.

For health insurance subscribers, Durbin-Watson’s statistic was 1.94, tolerance limits were between 0.39 and 0.92, and the VIF was between 1.09 and 2.56. Factors affecting the length of hospitalization in health insurers can be accounted for by 7.2% (*p* < .001). Factors affecting the length of hospital stay in health insurance subscribers were gender (*p* < .001), public pension status (*p* < .001), residential area (*p* < .001), administrative district (*p* < .010), economic activity (*p* < .001), income level (*p* < .001), perceived health status (*p* < .001), and disability status (*p* < .004).

Durbin-Watson’s statistic was 2.00, the tolerance limit was 0.62 to 0.99, and the VIF was between 1.01 and 1.59 for medical aid beneficiaries. Factors affecting the length of hospitalization for medical aid beneficiaries were accounted for by 3.1% (*p* < .001). Gender (*p* = .007) and perceived health status (*p* = .001) were identified as factors influencing the length of hospitalization of medical aid beneficiaries (Table 5).

## Discussion

Health insurance subscribers and medical aid beneficiaries differ in demographic and social characteristics. This study found that medical aid beneficiaries were more likely to have lack of a support system and had low economic activity participation rates compared with health insurance subscribers. More women were medical aid beneficiaries than health insurance subscribers, and more than half of medical aid beneficiaries were aged 60 or over. In addition, the proportion of medical aid beneficiaries living without a spouse was 72.5%, single households accounted for 38.5%, and the proportion of low-income households was 92.6%. In a study examining data from the Korea Medical Panel database [9], medical aid beneficiaries tended to have higher proportions of women, lower education levels, and more unemployment than health insurance subscribers, similar to the results of the present study.

Moreover, the rate of perceived poor health status was 28.8% higher in medical aid beneficiaries than in health insurance subscribers, and 80% of medical aid beneficiaries had chronic diseases in this study. These findings are consistent with a previous study by Lee [9]. Wada et al. [11] reported that people of lower socioeconomic status were more likely to have worse self-reported health. It is necessary to conduct further research exploring the reasons for the high rate of poor perceived poor health status and the higher prevalence of chronic diseases in medical aid beneficiaries.

The results of the present study indicated that the number of outpatient visits and the length of hospitalization was higher in medical aid beneficiaries than in health insurance subscribers. A study comparing medical expenses among the elderly [12] also found that medical aid beneficiaries had more treatment at outpatient clinics and longer length of hospitalization per capita than health insurance subscribers. In the comparative study by Lee [9], the number of hospitalization days of medical aid beneficiaries was found to be twice as high as that of health insurance subscribers, which is consistent with the results of the present study.

Among health insurance subscribers, it was found that subjects with lower incomes had higher frequencies of outpatient visits and more hospitalization days. Medical aid beneficiaries did not show any difference in the number of outpatient visits and the length of hospitalization according to their income level. This difference between health insurance subscribers and medical aid beneficiaries can be explained by the fact that medical aid beneficiaries have a lower income level in general compared with health insurance subscribers; 92.6% of the medical aid beneficiaries were low-income households in the present study.

In the present study, perceived poor health status and chronic disease were linked with longer length of hospitalization in both health insurance subscribers and medical aid beneficiaries. Perceived poor health status can lead to worse physical health, more social isolation, and emotional problems such as depression [13]. Therefore, self-evaluation of health status should be considered when developing health promotion programs for both health insurance subscribers and medical aid beneficiaries. Policies and health programs that also consider social support as well as physical health promotion are also needed. It is worth noting that, in the present study, in subjects who perceived their health condition as very poor, the length of hospitalization of the health insurance subscribers was 31.30 days and the length of hospitalization of the medical aid beneficiaries was 13.54 days (Table 4). This indicates that the length of hospitalization of health insurance subscribers was longer than those of medical aid beneficiaries. Low-income people tend to be more restricted in terms of access to health care and to be less able to afford to live healthier. They are also more likely to face financial barriers to paying deductibles, copayments, drug costs, and other health care costs [14]. Income and wealth appear to be linked to health. Therefore, more efforts should be made to understand how income drives health and to establish better policy by the government, community, and private sector to improve health and the management of costs of health care in medical aid beneficiaries.

Although age was identified as an influencing factor for the frequency of outpatient treatments in health insurance subscribers, it was not identified as an influencing factor in medical aid beneficiaries. Health insurance subscribers aged 70 and over visited outpatient clinics most frequently, whereas medical aid beneficiaries showed high use of medical services at all ages. Because there are few previous studies on this topic, it is difficult to make a direct comparison. However, the number of outpatient visits is thought to be high at all ages due to the demographic and social characteristics of 80% of health care recipients and 68.3% of people with disabilities. Therefore, it is thought that medical aid beneficiaries need to manage unnecessary medical care and reduce the number of outpatient visits, and this can be accomplished through chronic disease prevention and management programs and care for the disabled through all ages.

The characteristics that affect the number of outpatient visits in both health insurance and medical beneficiaries were household type, administrative district, and chronic disease. In cases of single households, the number of outpatient visits in both health insurance subscribers and medical aid beneficiaries increased. One previous study [15] found that living alone had an impact on health care use in older patients. Because members of single households tend to exhibit more passive health behaviours, it can be assumed that they are more likely to experience health problems, which may increase the prevalence of chronic diseases and result in health care use. For both health insurance subscribers and medical aid beneficiaries, the presence of chronic disease was a factor in the number of outpatient visits. This was confirmed in one previous study [16] in which overall medical expenses increase due to increased health care use, prescription drug use, and complications caused by chronic diseases. More focus should be place on chronic disease prevention through strengthening the education of the adolescent population and on chronic disease management through supporting chronic disease management programs or by expanding funding for the most common chronic diseases.

The government is constantly striving to ensure proper health care use and to prevent surges in medical expenses while strengthening the coverage of health insurance subscribers and beneficiaries. However, despite these efforts, health care use and medical expenses are increasing every year. A policy for providing customized health care services should be developed. In order to alleviate the effects of increasing health care use and medical expenses, it is necessary to expand preventive nursing services to better serve patients whose needs are affected by issues involving gender, household type, and perceived health status. It is also important to focus on health condition management for chronic diseases, the prevention of chronic diseases, and providing visits to the elderly who live alone. In addition, community care should focus more on providing specialized health care programs for low-educated, low-income, single-family households.

There is a limitations in this study. Different from our expectation, the demographic and socio-economic factors examined could only account for less than 20% of the explanation in correlation analysis. Because this study was based on the Korea Welfare Panel Study, variables such as property, commercial therapist, regional medical supply level, and distance from medical institutions were not considered. This is considered to be one of the reasons for the low explanatory power of health insurance subscribers and medical aid beneficiaries. The condition for the selection of medical aid beneficiaries are those who are not able to work or who are deemed difficult to work due to severe disability or who do not meet the median income in Kore. It is also possibility of combine effect of demographic and socio-economic factors. Further studies are needed to consider other factors and explore the combination effect of those factors. However, despite this limitation, this study examines the factors of health care use by considering socio-demographic, socio-economic, and health conditions in order to prevent bias of the study results due to socio-economic differences between health insurance subscribers and medical aid beneficiaries. The results of this study can be used to inform medical management policy such as expanding the level of medical insurance coverage or a guaranteed insurability.

## Conclusions

This study found that medical aid beneficiaries made more frequent outpatient visits and had more days of hospitalization than health insurance subscribers. Because the characteristics and the factors affecting health care use were different between health insurance subscribers and medical aid beneficiaries, policies and health programs that take into account the factors affecting the use of medical care will be required in order to promote appropriate health care use while enhancing services. More comprehensive research will be needed to examine the variables that have not been considered in this study, such as property, regional medical supply level, and distance from medical institutions.

## Supplementary information

**Additional file 1.**

## Data Availability

The data that support the findings of this study are available from the Korea Institute for Health and Social Affairs and the Seoul National University Institute for Social Welfare in Korea. Data are however available from the authors upon reasonable request and with permission of the Korea Institute for Health and Social Affairs and the Seoul National University Institute for Social Welfare in Korea.

## Abbreviations

OECD: Organisation for Economic Co-operation and Development
VIF: Variation inflation factor

## References

[1] OECD (2015). Health at a glancehttp://www.keepeek.com/Digital-Asset-Management/oecd/social-issues-migration-health/health-at-a-glance-2015_health_glance-2015-en#page27. Accessed on 29 October 2019.

[2] Korean Ministry of Health and Welfare. The health and welfare policy, starting in the first half of 2016, has changed. Accessed 28 Oct 2019. https://www.mohw.go.kr/react/al/sal0301vw.jsp?PAR_MENU_ID=04&MENU_ID=0403&CONT_SEQ=329246.

[3] Korean National Health Insurance Service database. Health insurance statistics in 2015. https://www.nhis.or.kr/bbs7/boards/B0074/17042?boardKey=29&sort=sequence&order=desc&rows=10&messageCategoryKey=&pageNumber=1&viewType=generic&targetType=12&targetKey=29&status=&period=&startdt=&enddt=&queryField=&query=. Accessed 28 Oct 2019.

[4] Shin H-W, Yeo J-Y, Kim J-H, Lee S-DK, Seong M-H (2014). The impact of supplier induced demand on increase in medical aid expenditure. Korean Acad Health Policy Manage.

[5] Koo JY, Yoo SH, Lee HJ, Son TY (2000). A comparison of the recognition and satisfaction for health care service between internal customer and external customer. Korean Acad Health Policy Manage.

[6] Rudramma J., Appasaheb W, Mallapur MD Socio-demographic factors influencing utilization of Antenatal Health Care Services in a rural area: a cross sectional study Int J Med Sci Public Health 2014; doi: 10.5455/ijmsph.2013.231220131.

[7] Saswata G. Socio-economic factors influencing utilisation of maternal health care in Uttar Pradesh: an analysis of NFHS-2 data. Soc Change. 2004. 10.1177/004908570403400405.

[8] Piovesan C, Ardenghi TM, Mendes FM, Agostini BA, Michel-Crosato E. Individual and contextual factors influencing dental health care utilization by preschool children: a multilevel analysis. Braz Oral Res. 2017. 10.1590/1807-3107bor-2017.vol31.0027. 10.1590/1807-3107BOR-2017.vol31.0027 28380090

[9] Lee H-J (2016). Healthcare utilization and out-of-pocket spending of medical aids recipients in South Korea: a propensity score matching with National Health Insurance participants. Korean J Health Econ Policy.

[10] Yu WS (2008). Problems and improvement measures of recent changes in medical benefits joining. Welf Trends.

[11] Wada K, Higuchi Y, Smith DR (2015). Socioeconomic status and self-reported health among middle-aged Japanese men: results from a nationwide longitudinal study. BMJ Open.

[12] Kim J-G (2010). A comparative study on the cause of medical expenditure for the elderly in national health insurance and medical assistance. Korean J Gerontol Soc Welf.

[13] Gunzelmann T, Hinz A, Brahler E (2006). Subjective health in older people. Psychosoc Med.

[14] Woolf SH, Aron L, Dubay L, Simon SM, Zimmerman E, Luk KX. How are income and wealth linked to health and longevity? Virginia Commonwealth University’s Center on Society and Health and the Urban Institute; 2015. https://www.urban.org/sites/default/files/publication/49116/2000178-How-are-Income-and-Wealth-Linked-to-Health-and-Longevity.pdf. Accessed 01 Nov 2019.

[15] Dreyer K, Steventon A, Fisher R, Deeny SR (2018). The association between living alone and health care utilisation in older adults: a retrospective cohort study of electronic health records from a London general practice. BMC Geriatr.

[16] Gotsadze G, Tang W, Shengelia N, Zoidze A (2017). Determinants analysis of outpatient service utilisation in Georgia: can the approach help inform benefit package design?. Health Res Policy Syst.

