# Supplementary material for: Factors influencing health care use by health insurance subscribers and medical aid beneficiaries: a study based on data from the Korea welfare panel study database
Source: BMC Public Health. 2020 Jul 20;20:1133. doi: 10.1186/s12889-020-09073-x (PMC7370477; doi:10.1186/s12889-020-09073-x)
Supplement: Supplementary file 1 — Additional file 1. [file 12889_2020_9073_MOESM1_ESM.docx]

Measurements of the study

| Measurement | Characteristics |
| --- | --- |
| Health care service use | Number of outpatient clinic visits, number of hospitalization days |
| Socio-demographic factors | Gender (‘man’ or ‘woman’), age (‘less than 20 years’, ‘21-29 years’, ‘30-39 years’, ‘40-49 years’, ‘50-59 years’, ’60-69 years’, or ‘70 or more years’), spouse (‘yes’ or ‘no’), household type (‘single family’ or ‘non-single family’), education (‘none’, ‘primary school’, ‘secondary school’, or ‘college or more’), private health insurance (‘joined’ or ‘did not join’), public pension (‘joined’ or ‘did not join’), residential area (‘city’ or ‘rural’), and administrative district |
| Socio-economic factors | Financial activity participation (‘yes’ or ‘no’) and low-income household status (‘yes’ or ‘no’) |
| Health status | Perceived health status (‘very good’, ‘good’, ‘neutral’, ‘poor’, or ‘very poor’), chronic disease (‘presence’ or ‘absence’), and disability (‘presence’ or ‘absence’). Chronic diseases were categorized by administration of medication or medical treatment. Absence of autism disorders, epilepsy disorders, mental disorders, kidney disorders, heart disorders, respiratory disorders, liver disorders, facial disorders, and ostomy disorders was classified as non-disabled. |
